# (4,4′,6,6′-Tetra-*tert*-butyl-2,2′-{[2-(di­methyl­amino)­ethyl]­nitrilo­bis­(methyl­ene)}diphenolato)dioxidomolyb­denum(VI) chloro­form monosolvate

**DOI:** 10.1107/S160053681104092X

**Published:** 2011-10-12

**Authors:** Xiangyang Lei, Nagasree Chelamalla

**Affiliations:** a Department of Chemistry & Biochemistry, Lamar University, Beaumont, TX 77710, USA

## Abstract

In the title compound, [Mo(C_34_H_54_N_2_O_2_)O_2_]·CHCl_3_, the molybdenum(VI) ion exhibits a *cis*-dioxide distorted octa­hedral geometry. Two anionic phenolate O-atom donors and two neutral N-atom donors of the ligand are *trans* and *cis*, respectively. The Mo=O bond lengths and the O=Mo=O bond angle are typical for six-coordinated dioxomolyb­denum(VI) complexes. The Mo—N bond lengths are longer than 2.30 Å, as expected for a *trans* O=Mo—N structure.

## Related literature

For molybdenum coordination complexes as catalysts, see: Wong *et al.* (2010 ▶); Rappe & Goddard (1982 ▶). For the synthesis of the ligand, see: Tshuva *et al.* (2001 ▶). For incorporation of the molybdenum center into redox enzymes, see: Tucci *et al.* (1998 ▶); Schultz *et al.* (1993 ▶). For spectroscopic and NMR data, see: Lehtonen *et al.* (2006 ▶). For related structures, see: Hinshaw *et al.* (1989 ▶); Lehtonen & Sillanpää (2005 ▶).
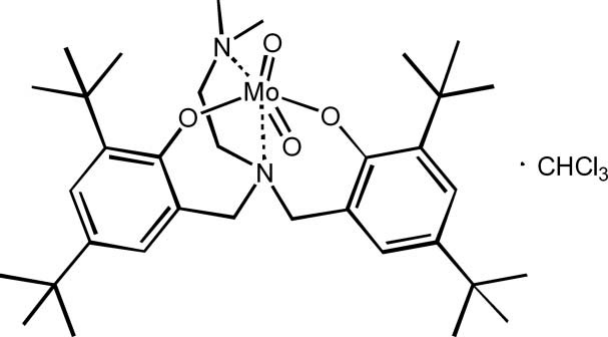


## Experimental

### 

#### Crystal data

[Mo(C_34_H_54_N_2_O_2_)O_2_]·CHCl_3_
*M* *_r_* = 770.10
Orthorhombic, 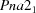

*a* = 24.3475 (10) Å
*b* = 13.9748 (6) Å
*c* = 11.0267 (4) Å
*V* = 3751.9 (3) Å^3^
*Z* = 4
Cu *K*α radiation
μ = 5.12 mm^−1^
*T* = 110 K
0.50 × 0.20 × 0.02 mm

#### Data collection

Bruker MWPC area-detector diffractometer
Absorption correction: multi-scan (*SADABS*; Bruker, 2008 ▶) *T*_min_ = 0.184, *T*_max_ = 0.904
79926 measured reflections
5548 independent reflections
5061 reflections with *I* > 2σ(*I*)
*R*_int_ = 0.081
θ_max_ = 60.0°

#### Refinement

*R*[*F* ^2^ > 2σ(*F* ^2^)] = 0.031
*wR*(*F* ^2^) = 0.078
*S* = 1.00
5548 reflections
421 parameters
1 restraint
H-atom parameters constrained
Δρ_max_ = 0.81 e Å^−3^
Δρ_min_ = −0.51 e Å^−3^
Absolute structure: Flack (1983 ▶), 2649 Friedel pairs
Flack parameter: 0.000 (9)

### 

Data collection: *FRAMBO* (Bruker, 1999 ▶); cell refinement: *FRAMBO*; data reduction: *SAINT* (Bruker, 2004 ▶); program(s) used to solve structure: *SHELXS97* (Sheldrick, 2008 ▶); program(s) used to refine structure: *SHELXL97* (Sheldrick, 2008 ▶); molecular graphics: *SHELXTL* (Sheldrick, 2008 ▶); software used to prepare material for publication: *SHELXTL*.

## Supplementary Material

Crystal structure: contains datablock(s) I, global. DOI: 10.1107/S160053681104092X/jj2100sup1.cif

Structure factors: contains datablock(s) I. DOI: 10.1107/S160053681104092X/jj2100Isup2.hkl

Additional supplementary materials:  crystallographic information; 3D view; checkCIF report

## Figures and Tables

**Fig. 1.**
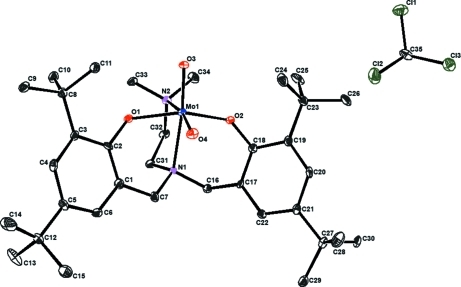
A view of the molecular structure. Ellipsoids are drawn at the 30% probability level. Hydrogen atoms were omitted for clarity.

## supplementary crystallographic information

### Comment

Molybdenum coordination complexes have attracted considerable attention because they can catalyze a variety of chemical reactions such as olefin epoxidation (Wong *et al.* 2010) and olefin metathesis (Rappe & Goddard, 1982) reactions. In addition, molybdenum is also a necessary element in diverse biological systems whereby the molybdenum center is incorporated into various redox enzymes such as DMSO reductase (Tucci *et al.* 1998) and xanthine oxidase (Schultz *et al.* 1993). A number of related dioxomolybdenum(VI) complexes with tetradentate ligands have been reported (Hinshaw *et al.* 1989; Lehtonen & Sillanpää, 2005).

While  the X-ray structure of the title compound is described here, its synthesis, IR, and ^1^H & ^13^C NMR data have been reported (Lehtonen *et al.* 2006).  The title complex contains one crystallographically unique molybdenum ion in a *cis*-dioxo distorted octahedral geometry. The aminobis(phenolate) moiety is coordinated to the MoO_2_^2+^ unit as a tripodal tetradentate ligand though two anionic phenolate oxygen donors (*trans* to each other) and two neutral nitrogen donors (*cis* to each other). The Mo=O bond lengths (1.702 (2) and 1.702 (3) Å for Mo=O3 and Mo=O4, respectively) and the O=Mo=O bond angle (108.33 (13)°) are typical for six-coordinated dioxomolybdenum(VI) complexes. The bond lengths of Mo—N1 and Mo—N2 are 2.392 (3) and 2.422 (3) Å, respectively, both of which are > 2.30 Å as expected for the *trans* O=Mo—N structure as well as a distorted octahedral geometry.

### Experimental

To a solution of 0.52 g (1.00 mmol) of 6,6'-(2-(dimethylamino)ethylazanediyl)bis(methylene)bis(2,4-di-*tert*-butylphenol) (Tshuva *et al.* 2001) in 10 ml of CH_2_Cl_2_ and 10 ml of CH_3_OH was added 0.33 g (1.06 mmol) of MoO_2_(acac)_2_. The resulting orange solution was stirred overnight at room temperature. The yellow solid (0.61 g) was collected by filtration and washed with cold methanol. Single crystals suitable for X-ray diffraction were obtained by recrystallization from CHCl_3_/hexanes.

### Refinement

All non-hydrogen atoms were refined with anisotropic thermal parameters. The hydrogen atoms bound to carbon atoms were placed in idealized positions and constrained to ride on their parent atoms, with d(C—H) = 0.95–1.00 Å, *U*_iso_(H) = 1.2*U*_eq_(C). The number of Friedel pairs used for absolute structure refinement is 2649.

### Crystal data

**Table:**

[Mo(C_34_H_54_N_2_O_2_)O_2_]·CHCl_3_	*F*(000) = 1616
*M**_r_* = 770.10	*D*_x_ = 1.363 Mg m^−^^3^
Orthorhombic, *P**n**a*2_1_	Cu *K*α radiation, λ = 1.54178 Å
Hall symbol: P 2c -2n	Cell parameters from 9879 reflections
*a* = 24.3475 (10) Å	θ = 3.6–62.7°
*b* = 13.9748 (6) Å	µ = 5.12 mm^−^^1^
*c* = 11.0267 (4) Å	*T* = 110 K
*V* = 3751.9 (3) Å^3^	Plate, yellow
*Z* = 4	0.50 × 0.20 × 0.02 mm

### Data collection

**Table:**

Bruker MWPC area-detector diffractometer	5548 independent reflections
Radiation source: fine-focus sealed tube	5061 reflections with *I* > 2σ(*I*)
graphite	*R*_int_ = 0.081
φ and ω scans	θ_max_ = 60.0°, θ_min_ = 4.8°
Absorption correction: multi-scan (*SADABS*; Bruker, 2008)	*h* = −27→27
*T*_min_ = 0.184, *T*_max_ = 0.904	*k* = −15→15
79926 measured reflections	*l* = −12→12

### Refinement

**Table:**

Refinement on *F*^2^	Secondary atom site location: difference Fourier map
Least-squares matrix: full	Hydrogen site location: inferred from neighbouring sites
*R*[*F*^2^ > 2σ(*F*^2^)] = 0.031	H-atom parameters constrained
*wR*(*F*^2^) = 0.078	*w* = 1/[σ^2^(*F*_o_^2^) + (0.054*P*)^2^] where *P* = (*F*_o_^2^ + 2*F*_c_^2^)/3
*S* = 1.00	(Δ/σ)_max_ = 0.001
5548 reflections	Δρ_max_ = 0.81 e Å^−^^3^
421 parameters	Δρ_min_ = −0.51 e Å^−^^3^
1 restraint	Absolute structure: Flack (1983), 2649 Friedel pairs
Primary atom site location: structure-invariant direct methods	Flack parameter: 0.000 (9)

### Special details

**Table:**

Geometry. All e.s.d.'s (except the e.s.d. in the dihedral angle between two l.s. planes) are estimated using the full covariance matrix. The cell e.s.d.'s are taken into account individually in the estimation of e.s.d.'s in distances, angles and torsion angles; correlations between e.s.d.'s in cell parameters are only used when they are defined by crystal symmetry. An approximate (isotropic) treatment of cell e.s.d.'s is used for estimating e.s.d.'s involving l.s. planes.
Refinement. Refinement of *F*^2^ against ALL reflections. The weighted *R*-factor *wR* and goodness of fit *S* are based on *F*^2^, conventional *R*-factors *R* are based on *F*, with *F* set to zero for negative *F*^2^. The threshold expression of *F*^2^ > 2σ(*F*^2^) is used only for calculating *R*-factors(gt) *etc.* and is not relevant to the choice of reflections for refinement. *R*-factors based on *F*^2^ are statistically about twice as large as those based on *F*, and *R*-factors based on ALL data will be even larger.

### Fractional atomic coordinates and isotropic or equivalent isotropic displacement parameters (Å^2^)

**Table:**

	*x*	*y*	*z*	*U*_iso_*/*U*_eq_	
Mo1	0.426810 (10)	0.838846 (17)	−0.50398 (3)	0.01403 (9)	
Cl1	0.59086 (5)	0.19093 (10)	−0.37928 (12)	0.0456 (3)	
Cl2	0.62313 (6)	0.38887 (9)	−0.37779 (18)	0.0700 (5)	
Cl3	0.70222 (5)	0.24265 (8)	−0.43262 (11)	0.0380 (3)	
O1	0.37192 (9)	0.93850 (17)	−0.4855 (3)	0.0161 (6)	
O2	0.48994 (10)	0.76272 (18)	−0.4523 (3)	0.0198 (6)	
O3	0.38528 (10)	0.74922 (18)	−0.5542 (2)	0.0195 (6)	
O4	0.45879 (11)	0.8885 (2)	−0.6260 (2)	0.0223 (6)	
N1	0.47151 (12)	0.9508 (2)	−0.3729 (3)	0.0135 (7)	
N2	0.39503 (13)	0.8003 (2)	−0.3016 (3)	0.0171 (7)	
C1	0.42256 (14)	1.0851 (3)	−0.4926 (5)	0.0205 (9)	
C2	0.37556 (14)	1.0319 (2)	−0.5192 (4)	0.0176 (9)	
C3	0.33115 (15)	1.0736 (3)	−0.5830 (3)	0.0159 (9)	
C4	0.33686 (16)	1.1685 (3)	−0.6165 (4)	0.0188 (9)	
H4	0.3075	1.1973	−0.6600	0.023*	
C5	0.38261 (16)	1.2249 (3)	−0.5909 (4)	0.0198 (9)	
C6	0.42495 (15)	1.1812 (3)	−0.5263 (4)	0.0202 (11)	
H6	0.4563	1.2179	−0.5047	0.024*	
C7	0.47454 (16)	1.0452 (3)	−0.4337 (4)	0.0199 (9)	
H7A	0.5032	1.0411	−0.4972	0.024*	
H7B	0.4873	1.0924	−0.3731	0.024*	
C8	0.27985 (16)	1.0155 (3)	−0.6168 (4)	0.0184 (9)	
C9	0.23863 (16)	1.0749 (3)	−0.6897 (4)	0.0230 (9)	
H9A	0.2064	1.0356	−0.7085	0.035*	
H9B	0.2558	1.0964	−0.7653	0.035*	
H9BC	0.2273	1.1306	−0.6419	0.035*	
C10	0.24975 (14)	0.9810 (3)	−0.5024 (5)	0.0239 (8)	
H10A	0.2181	0.9419	−0.5260	0.036*	
H10B	0.2371	1.0364	−0.4557	0.036*	
H10C	0.2748	0.9426	−0.4528	0.036*	
C11	0.29541 (17)	0.9285 (3)	−0.6952 (4)	0.0213 (9)	
H11A	0.2619	0.8972	−0.7245	0.032*	
H11B	0.3168	0.8831	−0.6465	0.032*	
H11C	0.3174	0.9498	−0.7645	0.032*	
C12	0.38648 (18)	1.3300 (3)	−0.6294 (4)	0.0252 (10)	
C13	0.3754 (2)	1.3935 (3)	−0.5203 (5)	0.0456 (14)	
H13A	0.3805	1.4606	−0.5432	0.068*	
H13B	0.4010	1.3771	−0.4549	0.068*	
H13C	0.3376	1.3836	−0.4924	0.068*	
C14	0.3457 (2)	1.3543 (3)	−0.7301 (5)	0.0437 (14)	
H14A	0.3502	1.4215	−0.7537	0.066*	
H14B	0.3082	1.3439	−0.7008	0.066*	
H14C	0.3526	1.3131	−0.8004	0.066*	
C15	0.4439 (2)	1.3524 (3)	−0.6790 (5)	0.0355 (12)	
H15A	0.4457	1.4198	−0.7031	0.053*	
H15B	0.4513	1.3118	−0.7496	0.053*	
H15C	0.4714	1.3399	−0.6161	0.053*	
C16	0.52969 (15)	0.9236 (3)	−0.3376 (4)	0.0192 (9)	
H16A	0.5495	0.9822	−0.3125	0.023*	
H16B	0.5281	0.8804	−0.2665	0.023*	
C17	0.56204 (14)	0.8750 (3)	−0.4370 (4)	0.0170 (9)	
C18	0.54237 (14)	0.7891 (3)	−0.4836 (4)	0.0178 (9)	
C19	0.57446 (15)	0.7309 (3)	−0.5608 (4)	0.0190 (9)	
C20	0.62607 (15)	0.7660 (3)	−0.5902 (4)	0.0176 (9)	
H20	0.6489	0.7279	−0.6407	0.021*	
C21	0.64651 (16)	0.8541 (3)	−0.5501 (4)	0.0174 (9)	
C22	0.61418 (15)	0.9075 (3)	−0.4715 (3)	0.0182 (10)	
H22	0.6275	0.9666	−0.4409	0.022*	
C23	0.55331 (17)	0.6345 (3)	−0.6097 (4)	0.0217 (9)	
C24	0.5097 (2)	0.6521 (3)	−0.7063 (5)	0.0348 (12)	
H24A	0.4975	0.5908	−0.7399	0.052*	
H24B	0.5252	0.6916	−0.7713	0.052*	
H24C	0.4783	0.6853	−0.6699	0.052*	
C25	0.52917 (19)	0.5717 (3)	−0.5084 (6)	0.0384 (10)	
H25A	0.5227	0.5070	−0.5395	0.058*	
H25B	0.4944	0.5992	−0.4806	0.058*	
H25C	0.5550	0.5688	−0.4404	0.058*	
C26	0.59983 (19)	0.5767 (3)	−0.6685 (5)	0.0329 (12)	
H26A	0.5860	0.5136	−0.6923	0.049*	
H26B	0.6300	0.5690	−0.6104	0.049*	
H26C	0.6132	0.6106	−0.7405	0.049*	
C27	0.70438 (16)	0.8849 (3)	−0.5894 (4)	0.0208 (9)	
C28	0.70892 (19)	0.8820 (4)	−0.7288 (4)	0.0339 (11)	
H28A	0.7468	0.8962	−0.7530	0.051*	
H28B	0.6841	0.9297	−0.7641	0.051*	
H28C	0.6987	0.8181	−0.7579	0.051*	
C29	0.71837 (17)	0.9870 (3)	−0.5474 (4)	0.0252 (10)	
H29A	0.7545	1.0052	−0.5788	0.038*	
H29B	0.7189	0.9892	−0.4586	0.038*	
H29C	0.6906	1.0316	−0.5781	0.038*	
C30	0.74679 (15)	0.8160 (3)	−0.5365 (4)	0.0239 (11)	
H30A	0.7837	0.8361	−0.5610	0.036*	
H30B	0.7397	0.7512	−0.5667	0.036*	
H30C	0.7442	0.8164	−0.4478	0.036*	
C31	0.43785 (16)	0.9580 (3)	−0.2602 (4)	0.0197 (9)	
H31A	0.4032	0.9923	−0.2783	0.024*	
H31B	0.4582	0.9952	−0.1985	0.024*	
C32	0.42489 (17)	0.8604 (3)	−0.2109 (4)	0.0196 (9)	
H32A	0.4595	0.8281	−0.1876	0.024*	
H32B	0.4020	0.8671	−0.1371	0.024*	
C33	0.33508 (16)	0.8180 (3)	−0.2903 (4)	0.0207 (9)	
H33A	0.3218	0.7915	−0.2133	0.031*	
H33B	0.3158	0.7872	−0.3578	0.031*	
H33C	0.3281	0.8871	−0.2922	0.031*	
C34	0.40419 (19)	0.6974 (3)	−0.2756 (4)	0.0273 (10)	
H34A	0.3912	0.6827	−0.1936	0.041*	
H34B	0.4435	0.6830	−0.2816	0.041*	
H34C	0.3839	0.6585	−0.3345	0.041*	
C35	0.64330 (19)	0.2714 (3)	−0.3474 (4)	0.0322 (11)	
H35	0.6526	0.2663	−0.2594	0.039*	

### Atomic displacement parameters (Å^2^)

**Table:**

	*U*^11^	*U*^22^	*U*^33^	*U*^12^	*U*^13^	*U*^23^
Mo1	0.01303 (14)	0.01394 (14)	0.01511 (14)	−0.00271 (11)	−0.00087 (19)	−0.00026 (19)
Cl1	0.0472 (7)	0.0482 (7)	0.0413 (8)	−0.0196 (6)	0.0070 (6)	−0.0063 (6)
Cl2	0.0577 (9)	0.0264 (7)	0.1258 (15)	0.0056 (6)	0.0374 (10)	0.0150 (8)
Cl3	0.0442 (7)	0.0329 (6)	0.0368 (6)	0.0014 (5)	0.0031 (6)	0.0034 (6)
O1	0.0132 (12)	0.0155 (12)	0.0196 (17)	−0.0015 (9)	−0.0039 (12)	0.0029 (13)
O2	0.0140 (13)	0.0168 (14)	0.0287 (15)	−0.0036 (11)	0.0027 (12)	−0.0061 (12)
O3	0.0168 (13)	0.0163 (14)	0.0253 (14)	−0.0043 (11)	−0.0024 (12)	−0.0040 (12)
O4	0.0237 (16)	0.0274 (16)	0.0160 (14)	−0.0045 (13)	0.0041 (13)	0.0004 (13)
N1	0.0130 (16)	0.0124 (16)	0.0151 (16)	−0.0018 (13)	0.0032 (15)	−0.0010 (14)
N2	0.0199 (18)	0.0163 (17)	0.0150 (17)	−0.0020 (14)	0.0007 (15)	0.0021 (15)
C1	0.0195 (18)	0.0191 (18)	0.023 (2)	0.0011 (15)	0.000 (2)	0.004 (2)
C2	0.0219 (18)	0.0100 (17)	0.021 (3)	−0.0029 (13)	0.002 (2)	−0.005 (2)
C3	0.012 (2)	0.019 (2)	0.017 (2)	0.0016 (16)	0.0024 (17)	−0.0024 (18)
C4	0.017 (2)	0.023 (2)	0.016 (2)	0.0072 (17)	0.0020 (18)	0.0049 (18)
C5	0.026 (2)	0.015 (2)	0.019 (2)	0.0030 (17)	0.0015 (19)	0.0021 (18)
C6	0.0181 (18)	0.0177 (19)	0.025 (3)	−0.0039 (15)	−0.0005 (19)	0.0019 (19)
C7	0.020 (2)	0.015 (2)	0.024 (2)	0.0032 (16)	0.002 (2)	0.0037 (18)
C8	0.020 (2)	0.019 (2)	0.016 (2)	−0.0001 (17)	−0.0019 (18)	−0.0014 (18)
C9	0.017 (2)	0.026 (2)	0.027 (2)	−0.0010 (18)	−0.0070 (19)	0.001 (2)
C10	0.0185 (18)	0.0254 (19)	0.0278 (19)	0.0013 (15)	0.004 (3)	0.004 (3)
C11	0.019 (2)	0.024 (2)	0.021 (2)	0.0018 (18)	−0.0032 (18)	−0.0062 (19)
C12	0.028 (2)	0.013 (2)	0.034 (2)	0.0008 (17)	0.000 (2)	0.0013 (19)
C13	0.078 (4)	0.018 (2)	0.041 (3)	0.012 (2)	0.005 (3)	−0.001 (3)
C14	0.054 (3)	0.025 (3)	0.052 (3)	−0.006 (2)	−0.015 (3)	0.018 (2)
C15	0.036 (3)	0.024 (3)	0.047 (3)	−0.002 (2)	0.003 (2)	0.010 (2)
C16	0.016 (2)	0.019 (2)	0.022 (2)	−0.0004 (16)	−0.0020 (18)	−0.0059 (18)
C17	0.0076 (19)	0.020 (2)	0.023 (2)	0.0022 (16)	−0.0013 (17)	−0.0043 (19)
C18	0.0163 (19)	0.0179 (19)	0.019 (3)	0.0002 (15)	−0.0003 (18)	−0.0011 (18)
C19	0.016 (2)	0.022 (2)	0.0189 (19)	0.0057 (17)	−0.0060 (18)	−0.0044 (18)
C20	0.016 (2)	0.020 (2)	0.016 (2)	0.0086 (17)	−0.0001 (17)	−0.0037 (18)
C21	0.014 (2)	0.021 (2)	0.0174 (19)	0.0063 (17)	−0.0048 (16)	0.0043 (16)
C22	0.0174 (19)	0.0164 (19)	0.021 (3)	0.0004 (16)	−0.0033 (16)	−0.0059 (16)
C23	0.022 (2)	0.017 (2)	0.026 (2)	0.0048 (17)	−0.008 (2)	−0.0032 (19)
C24	0.034 (3)	0.033 (3)	0.037 (3)	0.013 (2)	−0.016 (2)	−0.019 (2)
C25	0.053 (3)	0.019 (2)	0.043 (3)	−0.0036 (18)	0.003 (4)	−0.012 (3)
C26	0.032 (3)	0.020 (2)	0.047 (3)	0.007 (2)	−0.010 (2)	−0.017 (2)
C27	0.014 (2)	0.028 (2)	0.020 (2)	0.0030 (17)	0.0020 (18)	0.0025 (19)
C28	0.026 (2)	0.052 (3)	0.024 (3)	0.001 (2)	0.007 (2)	0.004 (2)
C29	0.018 (2)	0.026 (2)	0.031 (2)	0.0006 (17)	0.0062 (18)	0.0032 (19)
C30	0.0125 (19)	0.028 (2)	0.031 (3)	0.0056 (16)	0.0024 (18)	−0.0025 (19)
C31	0.017 (2)	0.021 (2)	0.021 (2)	−0.0024 (17)	0.0019 (18)	−0.0041 (18)
C32	0.019 (2)	0.025 (2)	0.014 (2)	−0.0050 (18)	0.0008 (17)	0.0066 (18)
C33	0.015 (2)	0.025 (2)	0.022 (2)	−0.0053 (17)	0.0033 (18)	0.0001 (19)
C34	0.037 (3)	0.017 (2)	0.028 (2)	0.001 (2)	−0.003 (2)	0.009 (2)
C35	0.050 (3)	0.021 (2)	0.026 (2)	−0.005 (2)	−0.001 (2)	0.000 (2)

### Geometric parameters (Å, °)

**Table:**

Mo1—O3	1.702 (2)	C15—H15B	0.9800
Mo1—O4	1.702 (3)	C15—H15C	0.9800
Mo1—O1	1.941 (2)	C16—C17	1.512 (5)
Mo1—O2	1.954 (3)	C16—H16A	0.9900
Mo1—N1	2.392 (3)	C16—H16B	0.9900
Mo1—N2	2.422 (3)	C17—C18	1.391 (5)
Cl1—C35	1.737 (4)	C17—C22	1.401 (5)
Cl2—C35	1.746 (4)	C18—C19	1.413 (6)
Cl3—C35	1.761 (5)	C19—C20	1.387 (5)
O1—C2	1.360 (4)	C19—C23	1.540 (6)
O2—C18	1.373 (4)	C20—C21	1.400 (5)
N1—C7	1.482 (5)	C20—H20	0.9500
N1—C31	1.492 (5)	C21—C22	1.388 (5)
N1—C16	1.517 (5)	C21—C27	1.536 (5)
N2—C34	1.483 (5)	C22—H22	0.9500
N2—C33	1.486 (5)	C23—C24	1.525 (6)
N2—C32	1.495 (5)	C23—C26	1.535 (6)
C1—C6	1.395 (5)	C23—C25	1.538 (7)
C1—C2	1.396 (5)	C24—H24A	0.9800
C1—C7	1.528 (6)	C24—H24B	0.9800
C2—C3	1.416 (5)	C24—H24C	0.9800
C3—C4	1.384 (5)	C25—H25A	0.9800
C3—C8	1.536 (5)	C25—H25B	0.9800
C4—C5	1.393 (6)	C25—H25C	0.9800
C4—H4	0.9500	C26—H26A	0.9800
C5—C6	1.394 (6)	C26—H26B	0.9800
C5—C12	1.531 (5)	C26—H26C	0.9800
C6—H6	0.9500	C27—C30	1.528 (5)
C7—H7A	0.9900	C27—C29	1.537 (6)
C7—H7B	0.9900	C27—C28	1.541 (6)
C8—C9	1.531 (5)	C28—H28A	0.9800
C8—C10	1.536 (6)	C28—H28B	0.9800
C8—C11	1.539 (5)	C28—H28C	0.9800
C9—H9A	0.9800	C29—H29A	0.9800
C9—H9B	0.9800	C29—H29B	0.9800
C9—H9BC	0.9800	C29—H29C	0.9800
C10—H10A	0.9800	C30—H30A	0.9800
C10—H10B	0.9800	C30—H30B	0.9800
C10—H10C	0.9800	C30—H30C	0.9800
C11—H11A	0.9800	C31—C32	1.501 (6)
C11—H11B	0.9800	C31—H31A	0.9900
C11—H11C	0.9800	C31—H31B	0.9900
C12—C13	1.519 (7)	C32—H32A	0.9900
C12—C14	1.528 (7)	C32—H32B	0.9900
C12—C15	1.534 (7)	C33—H33A	0.9800
C13—H13A	0.9800	C33—H33B	0.9800
C13—H13B	0.9800	C33—H33C	0.9800
C13—H13C	0.9800	C34—H34A	0.9800
C14—H14A	0.9800	C34—H34B	0.9800
C14—H14B	0.9800	C34—H34C	0.9800
C14—H14C	0.9800	C35—H35	1.0000
C15—H15A	0.9800		
			
O3—Mo1—O4	108.33 (13)	C17—C16—H16A	108.7
O3—Mo1—O1	98.81 (11)	N1—C16—H16A	108.7
O4—Mo1—O1	96.06 (12)	C17—C16—H16B	108.7
O3—Mo1—O2	99.29 (11)	N1—C16—H16B	108.7
O4—Mo1—O2	95.31 (12)	H16A—C16—H16B	107.6
O1—Mo1—O2	154.36 (12)	C18—C17—C22	119.4 (3)
O3—Mo1—N1	161.44 (12)	C18—C17—C16	118.4 (3)
O4—Mo1—N1	90.18 (12)	C22—C17—C16	121.6 (3)
O1—Mo1—N1	77.33 (10)	O2—C18—C17	117.3 (3)
O2—Mo1—N1	79.73 (10)	O2—C18—C19	120.7 (3)
O3—Mo1—N2	86.91 (12)	C17—C18—C19	121.9 (3)
O4—Mo1—N2	164.76 (12)	C20—C19—C18	116.0 (4)
O1—Mo1—N2	80.96 (11)	C20—C19—C23	122.0 (3)
O2—Mo1—N2	82.04 (11)	C18—C19—C23	122.0 (3)
N1—Mo1—N2	74.57 (10)	C19—C20—C21	124.0 (4)
C2—O1—Mo1	128.0 (2)	C19—C20—H20	118.0
C18—O2—Mo1	120.8 (2)	C21—C20—H20	118.0
C7—N1—C31	110.1 (3)	C22—C21—C20	117.9 (4)
C7—N1—C16	107.0 (3)	C22—C21—C27	123.1 (4)
C31—N1—C16	108.4 (3)	C20—C21—C27	118.9 (3)
C7—N1—Mo1	109.3 (2)	C21—C22—C17	120.6 (4)
C31—N1—Mo1	107.3 (2)	C21—C22—H22	119.7
C16—N1—Mo1	114.6 (2)	C17—C22—H22	119.7
C34—N2—C33	107.0 (3)	C24—C23—C26	107.7 (4)
C34—N2—C32	110.0 (3)	C24—C23—C25	109.5 (4)
C33—N2—C32	109.1 (3)	C26—C23—C25	106.8 (3)
C34—N2—Mo1	110.2 (2)	C24—C23—C19	109.7 (3)
C33—N2—Mo1	110.8 (2)	C26—C23—C19	111.2 (3)
C32—N2—Mo1	109.6 (2)	C25—C23—C19	111.9 (4)
C6—C1—C2	119.4 (4)	C23—C24—H24A	109.5
C6—C1—C7	115.5 (3)	C23—C24—H24B	109.5
C2—C1—C7	125.0 (3)	H24A—C24—H24B	109.5
O1—C2—C1	120.5 (3)	C23—C24—H24C	109.5
O1—C2—C3	118.8 (3)	H24A—C24—H24C	109.5
C1—C2—C3	120.7 (3)	H24B—C24—H24C	109.5
C4—C3—C2	116.8 (3)	C23—C25—H25A	109.5
C4—C3—C8	121.6 (3)	C23—C25—H25B	109.5
C2—C3—C8	121.6 (3)	H25A—C25—H25B	109.5
C3—C4—C5	124.6 (4)	C23—C25—H25C	109.5
C3—C4—H4	117.7	H25A—C25—H25C	109.5
C5—C4—H4	117.7	H25B—C25—H25C	109.5
C4—C5—C6	116.5 (4)	C23—C26—H26A	109.5
C4—C5—C12	122.4 (4)	C23—C26—H26B	109.5
C6—C5—C12	121.1 (4)	H26A—C26—H26B	109.5
C5—C6—C1	121.9 (4)	C23—C26—H26C	109.5
C5—C6—H6	119.1	H26A—C26—H26C	109.5
C1—C6—H6	119.1	H26B—C26—H26C	109.5
N1—C7—C1	118.5 (3)	C30—C27—C21	109.6 (3)
N1—C7—H7A	107.7	C30—C27—C29	108.7 (3)
C1—C7—H7A	107.7	C21—C27—C29	112.2 (3)
N1—C7—H7B	107.7	C30—C27—C28	108.4 (3)
C1—C7—H7B	107.7	C21—C27—C28	109.9 (3)
H7A—C7—H7B	107.1	C29—C27—C28	108.0 (4)
C9—C8—C10	106.8 (3)	C27—C28—H28A	109.5
C9—C8—C3	111.9 (3)	C27—C28—H28B	109.5
C10—C8—C3	110.8 (3)	H28A—C28—H28B	109.5
C9—C8—C11	107.1 (3)	C27—C28—H28C	109.5
C10—C8—C11	109.3 (3)	H28A—C28—H28C	109.5
C3—C8—C11	110.7 (3)	H28B—C28—H28C	109.5
C8—C9—H9A	109.5	C27—C29—H29A	109.5
C8—C9—H9B	109.5	C27—C29—H29B	109.5
H9A—C9—H9B	109.5	H29A—C29—H29B	109.5
C8—C9—H9BC	109.5	C27—C29—H29C	109.5
H9A—C9—H9BC	109.5	H29A—C29—H29C	109.5
H9B—C9—H9BC	109.5	H29B—C29—H29C	109.5
C8—C10—H10A	109.5	C27—C30—H30A	109.5
C8—C10—H10B	109.5	C27—C30—H30B	109.5
H10A—C10—H10B	109.5	H30A—C30—H30B	109.5
C8—C10—H10C	109.5	C27—C30—H30C	109.5
H10A—C10—H10C	109.5	H30A—C30—H30C	109.5
H10B—C10—H10C	109.5	H30B—C30—H30C	109.5
C8—C11—H11A	109.5	N1—C31—C32	110.8 (3)
C8—C11—H11B	109.5	N1—C31—H31A	109.5
H11A—C11—H11B	109.5	C32—C31—H31A	109.5
C8—C11—H11C	109.5	N1—C31—H31B	109.5
H11A—C11—H11C	109.5	C32—C31—H31B	109.5
H11B—C11—H11C	109.5	H31A—C31—H31B	108.1
C13—C12—C14	109.3 (4)	N2—C32—C31	111.7 (3)
C13—C12—C5	109.2 (4)	N2—C32—H32A	109.3
C14—C12—C5	112.0 (4)	C31—C32—H32A	109.3
C13—C12—C15	109.0 (4)	N2—C32—H32B	109.3
C14—C12—C15	106.7 (4)	C31—C32—H32B	109.3
C5—C12—C15	110.5 (3)	H32A—C32—H32B	107.9
C12—C13—H13A	109.5	N2—C33—H33A	109.5
C12—C13—H13B	109.5	N2—C33—H33B	109.5
H13A—C13—H13B	109.5	H33A—C33—H33B	109.5
C12—C13—H13C	109.5	N2—C33—H33C	109.5
H13A—C13—H13C	109.5	H33A—C33—H33C	109.5
H13B—C13—H13C	109.5	H33B—C33—H33C	109.5
C12—C14—H14A	109.5	N2—C34—H34A	109.5
C12—C14—H14B	109.5	N2—C34—H34B	109.5
H14A—C14—H14B	109.5	H34A—C34—H34B	109.5
C12—C14—H14C	109.5	N2—C34—H34C	109.5
H14A—C14—H14C	109.5	H34A—C34—H34C	109.5
H14B—C14—H14C	109.5	H34B—C34—H34C	109.5
C12—C15—H15A	109.5	Cl1—C35—Cl2	111.3 (3)
C12—C15—H15B	109.5	Cl1—C35—Cl3	110.1 (2)
H15A—C15—H15B	109.5	Cl2—C35—Cl3	110.0 (2)
C12—C15—H15C	109.5	Cl1—C35—H35	108.5
H15A—C15—H15C	109.5	Cl2—C35—H35	108.5
H15B—C15—H15C	109.5	Cl3—C35—H35	108.5
C17—C16—N1	114.4 (3)		
			
O3—Mo1—O1—C2	−136.2 (3)	C16—N1—C7—C1	172.9 (4)
O4—Mo1—O1—C2	−26.5 (3)	Mo1—N1—C7—C1	48.2 (4)
O2—Mo1—O1—C2	89.4 (4)	C6—C1—C7—N1	167.6 (4)
N1—Mo1—O1—C2	62.3 (3)	C2—C1—C7—N1	−15.7 (7)
N2—Mo1—O1—C2	138.4 (3)	C4—C3—C8—C9	0.4 (5)
O3—Mo1—O2—C18	133.7 (3)	C2—C3—C8—C9	−177.7 (4)
O4—Mo1—O2—C18	24.1 (3)	C4—C3—C8—C10	−118.6 (4)
O1—Mo1—O2—C18	−91.9 (3)	C2—C3—C8—C10	63.2 (5)
N1—Mo1—O2—C18	−65.1 (3)	C4—C3—C8—C11	119.9 (4)
N2—Mo1—O2—C18	−140.8 (3)	C2—C3—C8—C11	−58.3 (5)
O3—Mo1—N1—C7	−138.1 (3)	C4—C5—C12—C13	102.5 (5)
O4—Mo1—N1—C7	37.9 (2)	C6—C5—C12—C13	−76.6 (5)
O1—Mo1—N1—C7	−58.3 (2)	C4—C5—C12—C14	−18.7 (6)
O2—Mo1—N1—C7	133.3 (2)	C6—C5—C12—C14	162.1 (4)
N2—Mo1—N1—C7	−142.2 (2)	C4—C5—C12—C15	−137.6 (4)
O3—Mo1—N1—C31	−18.7 (5)	C6—C5—C12—C15	43.3 (6)
O4—Mo1—N1—C31	157.3 (2)	C7—N1—C16—C17	−85.1 (4)
O1—Mo1—N1—C31	61.2 (2)	C31—N1—C16—C17	156.2 (3)
O2—Mo1—N1—C31	−107.3 (2)	Mo1—N1—C16—C17	36.3 (4)
N2—Mo1—N1—C31	−22.8 (2)	N1—C16—C17—C18	−60.3 (5)
O3—Mo1—N1—C16	101.8 (4)	N1—C16—C17—C22	128.1 (4)
O4—Mo1—N1—C16	−82.2 (3)	Mo1—O2—C18—C17	64.7 (4)
O1—Mo1—N1—C16	−178.4 (3)	Mo1—O2—C18—C19	−114.9 (3)
O2—Mo1—N1—C16	13.2 (2)	C22—C17—C18—O2	−176.6 (3)
N2—Mo1—N1—C16	97.7 (3)	C16—C17—C18—O2	11.6 (5)
O3—Mo1—N2—C34	54.6 (3)	C22—C17—C18—C19	3.0 (6)
O4—Mo1—N2—C34	−126.1 (5)	C16—C17—C18—C19	−168.8 (4)
O1—Mo1—N2—C34	154.0 (3)	O2—C18—C19—C20	177.7 (3)
O2—Mo1—N2—C34	−45.2 (3)	C17—C18—C19—C20	−1.9 (6)
N1—Mo1—N2—C34	−126.7 (3)	O2—C18—C19—C23	−2.1 (6)
O3—Mo1—N2—C33	−63.7 (2)	C17—C18—C19—C23	178.4 (4)
O4—Mo1—N2—C33	115.6 (5)	C18—C19—C20—C21	−1.2 (6)
O1—Mo1—N2—C33	35.8 (2)	C23—C19—C20—C21	178.5 (4)
O2—Mo1—N2—C33	−163.5 (2)	C19—C20—C21—C22	3.1 (6)
N1—Mo1—N2—C33	115.0 (2)	C19—C20—C21—C27	−179.9 (4)
O3—Mo1—N2—C32	175.9 (3)	C20—C21—C22—C17	−2.0 (6)
O4—Mo1—N2—C32	−4.9 (6)	C27—C21—C22—C17	−178.8 (4)
O1—Mo1—N2—C32	−84.7 (2)	C18—C17—C22—C21	−1.0 (6)
O2—Mo1—N2—C32	76.0 (2)	C16—C17—C22—C21	170.6 (4)
N1—Mo1—N2—C32	−5.4 (2)	C20—C19—C23—C24	−106.9 (5)
Mo1—O1—C2—C1	−46.0 (6)	C18—C19—C23—C24	72.9 (5)
Mo1—O1—C2—C3	133.2 (3)	C20—C19—C23—C26	12.1 (5)
C6—C1—C2—O1	−179.0 (4)	C18—C19—C23—C26	−168.2 (4)
C7—C1—C2—O1	4.5 (7)	C20—C19—C23—C25	131.4 (4)
C6—C1—C2—C3	1.9 (7)	C18—C19—C23—C25	−48.9 (5)
C7—C1—C2—C3	−174.7 (4)	C22—C21—C27—C30	112.4 (4)
O1—C2—C3—C4	−179.4 (4)	C20—C21—C27—C30	−64.4 (5)
C1—C2—C3—C4	−0.2 (6)	C22—C21—C27—C29	−8.4 (5)
O1—C2—C3—C8	−1.2 (6)	C20—C21—C27—C29	174.7 (3)
C1—C2—C3—C8	178.0 (4)	C22—C21—C27—C28	−128.7 (4)
C2—C3—C4—C5	−0.5 (6)	C20—C21—C27—C28	54.5 (5)
C8—C3—C4—C5	−178.8 (4)	C7—N1—C31—C32	168.7 (3)
C3—C4—C5—C6	−0.4 (6)	C16—N1—C31—C32	−74.5 (4)
C3—C4—C5—C12	−179.5 (4)	Mo1—N1—C31—C32	49.8 (3)
C4—C5—C6—C1	2.1 (6)	C34—N2—C32—C31	155.2 (3)
C12—C5—C6—C1	−178.7 (4)	C33—N2—C32—C31	−87.7 (4)
C2—C1—C6—C5	−2.9 (7)	Mo1—N2—C32—C31	33.8 (4)
C7—C1—C6—C5	174.0 (4)	N1—C31—C32—N2	−58.0 (4)
C31—N1—C7—C1	−69.4 (5)		
